# Snake Genome Sequencing: Results and Future Prospects

**DOI:** 10.3390/toxins8120360

**Published:** 2016-12-01

**Authors:** Harald M. I. Kerkkamp, R. Manjunatha Kini, Alexey S. Pospelov, Freek J. Vonk, Christiaan V. Henkel, Michael K. Richardson

**Affiliations:** 1 Institute of Biology, University of Leiden, Leiden 2300 RA, The Netherlands; h.m.i.kerkkamp@biology.leidenuniv.nl (H.M.I.K.); henkel.c@hsleiden.nl (C.V.H.); 2 Department of Biological Science, National University of Singapore, Singapore 117543, Singapore; dbskinim@nus.edu.sg; 3 Department of Biosciences and Neuroscience Center, University of Helsinki, Helsinki 00014, Finland; apospelo@mappi.helsinki.fi; 4 Naturalis Biodiversity Center, Darwinweg 2, Leiden 2333 CR, The Netherlands; freek.vonk@naturalis.nl

**Keywords:** snake, genome, genomics, king cobra, reptile, Malayan pit viper

## Abstract

Snake genome sequencing is in its infancy—very much behind the progress made in sequencing the genomes of humans, model organisms and pathogens relevant to biomedical research, and agricultural species. We provide here an overview of some of the snake genome projects in progress, and discuss the biological findings, with special emphasis on toxinology, from the small number of draft snake genomes already published. We discuss the future of snake genomics, pointing out that new sequencing technologies will help overcome the problem of repetitive sequences in assembling snake genomes. Genome sequences are also likely to be valuable in examining the clustering of toxin genes on the chromosomes, in designing recombinant antivenoms and in studying the epigenetic regulation of toxin gene expression.

## 1. Introduction

The sequencing of animal genomes is uncovering a treasure trove of biological information. Genomes can be defined in various ways. Functional definitions based on concepts of information-encoding and transfer tend to ignore the role of extra-genomic (epigenetic) mechanisms in these processes [1]. Therefore, we shall simply assume the genome to comprise the nucleotide sequence of all nuclear and mitochondrial DNA of an organism. The genome may be sequenced in its entirety via whole genome sequencing [2,3]. It may be more practical for some research questions to sequence only the region of interest, using a ‘targeted capture’ approach [4]. Targeted approaches include the selective sequencing of bacterial artificial chromosome (BAC) libraries [5].

Genome sequencing has tended to focus on *Homo sapiens* and there are reportedly plans to sequence 2 million human genomes for biomedical research objectives including personalized medicine [6]. Further, the genomes of many animal species used as models in biomedical research, or reared in agriculture, have also been sequenced. The genomes of non-model species have received far less attention although there are plans to sequence many thousands of vertebrate genome in the near future [7].

### 1.1. Why Snakes Are Interesting

Snake genomics is a neglected topic, as can be seen by the relatively modest number of published genomes and projects in the pipeline (Table 1). Nonetheless, it is a topic that is attracting increasing interest from a biologists in several sub-disciplines [8]. This interest in snake genomes stems from the medical importance of snakebite in many developing countries [9], the potential for finding novel drugs and other bioactive compounds in venoms [10] and, from the perspective of fundamental research, the extraordinary array of evolutionary novelties found in snakes [11,12].

Snakes (Serpentes) are represented by around 3000 extant species [20]. They show a suite of adaptations common to many lineages of vertebrates that have independently evolved long, thin bodies. This suite includes limb reduction or loss, axial elongation, increase in vertebral count and asymmetry of paired viscera. Extant snakes have completely lost all traces of the forelimb and pectoral girdle. In most species there is also loss of the hindlimb and pelvic girdle [11]. Exceptions include the femoral and pelvic girdle remnants found on each side in Leptotyphlopidae (reviewed in Ref. [21]); and the single pelvic element on each side in Typhlopidae [22]. Pelvic vestiges are also present in Aniliidae, Cylindrophiidae and Anomochilidae; and in boas and pythons. There are both pelvic and femoral vestiges, the latter often tipped with a horny spur [22,23]. Compared to ancestral squamates, snakes show elongation of the primary axis with a high vertebral number and poor demarcation of the vertebral regions [24]. The left lung is reduced in size or absent [25].

Other adaptations include jaw modifications and metabolic adaptations associated with swallowing prey whole [2]; the presence of a venom delivery system [26], consisting of the venom glands and fangs; and heat-sensing “pit organs”. Venom delivery systems are found in approximately 600 species in the Elapidae, Viperidae, Colubridae and Atractaspididae [27]. Heat-sensitive pit organs are represented by the loreal pit of Crotalinae [28], and the labial pits of some pythons and boas [29].

In the context of this special journal issue, the most relevant of these adaptations is venom, and the peptide or protein toxins that it contains. Venom can be defined as any glandular secretion produced by a metazoan that can be introduced into the tissues of another animal through a puncture (inflicted by the venomous animal for that purpose) and which incapacitates prey or deters attackers by virtue of its potent bioactivity [30]. Venom, and an associated venom delivery system (a gland connected to a puncturing device), has evolved independently in many animal clades [31,32].

### 1.2. What Genomes Can Tell Us

As has been pointed out [33], toxin evolution has been studied for many years using traditional sequencing and proteomics approaches; but new tools for genomics, transcriptomics and proteomics are greatly advancing the field. For biomedical research in general, there are many advantages in having genomic sequence data and we now summarize just a few of these advantages. A whole genome sequence allows, in principle, the prediction of all translated genes (the exome) by means of ab initio gene prediction algorithms [34] and homology searches using reference sequences [35]. Because of the paucity of genome sequences and the apparent frequent duplication of toxin-encoding genes, the use of transcriptome data from the same species, or one closely related, makes this task easier. The genes predicted may include genes for translated proteins as well as microRNAs (mRNAs) and other non-coding genes.

Predicted gene sequences can then be used in a host of applications and analyses, ranging from the design of probes for in situ hybridization [26], to searches for genes under positive or negative selection, as inferred by the *d*_N_/*d*_S_ ratio [36]. The latter analyses have shown that multiple genes are under selection in snakes, or in clades within the snakes, including some venom toxin genes [3] and developmental genes possibly connected to development of the serpentiform body plan [2].

With genome sequences, evolutionary gene loss can be more confidently asserted than by looking at the transcriptome alone. Hypotheses about gene loss, or the degeneration of functional genes into pseudogenes, can be more easily tested because non-coding pseudogenes can be identified in the genome sequence on the basis of sequence homology or synteny [37]. Synteny refers to the location of loci on the same chromosome, or the order and orientation of neighboring genes, especially when compared across species [37].

Analysis of genome sequences shows that several visual pigment genes have been lost in snakes compared with other squamates [2]. This may be related to the putative fossorial (burrowing) lifestyle of an ancestral snake which might have had reduced eyes [38]. Genomics also reveals that some neurotoxin genes have been lost in the lineage leading to the Western and Eastern diamond-backed rattlesnakes (*Crotalus atrox* and *C. adamanteus*, respectively) [5].

Using genome sequences, it is possible to look for candidate regulatory regions; this in turn may allow genomic regulatory blocks to be identified [39]. In the context of toxinology, it will be especially interesting to examine whether duplicated toxin genes of the same toxin family are clustered [5] and functionally part of a common regulatory landscape—in a way analogous, perhaps, to the well-studied *hox* developmental genes [16]. Genome sequences allow the identification of structural variations, including inversions, insertions, deletions and tandem duplications and other large rearrangements [40]. It is also possible to look for transposable elements and other repetitive sequences [41].

Genomic data can be used in phylogeny reconstruction (which is one aspect of the discipline phylogenomics) although this endeavor is not without difficulties [37,42]. One such difficulty is that the evolution of nucleotide sequences effectively overwrites the ancestral sequence making homologies (orthologues) more difficult to identify [37,42]. Horizontal gene transfer (between species), gene loss and genome duplications [43] can further obscure the phylogenetic relationships among species (reviewed in Ref. [37]).

### 1.3. Aims and Objectives of This Review

Our aim here is to review some of the biological results yielded, to date, by snake genomes; and to consider some of the research questions that may one day be solved by the analysis of snake genomes. We will focus mainly on the evolution of venom toxins, but discuss also some questions related to snake morphological and physiological adaptations that are being illuminated by genomics.

## 2. Status of Snake Genome Sequencing Projects

The sequencing of snake genomes is very much in its infancy. The first draft genomes of snakes to be published were those of the Boa constrictor (*Boa constrictor*) [13,14], Burmese python (*Python molurus bivittatus*) [2] and the king cobra (*Ophiophagus hannah*) [3], followed by a high coverage (238 ×) assembly of the first viper genome (*Crotalus mitchellii*) [18]. Some key data on the first two of these draft genomes are summarized in Table 2. The status of some other snake genome projects known to us, including studies based on targeted capture, is summarized in Table 1. As can be seen, the genome sizes of the Burmese python [2] and king cobra [3] are 1.44 and 1.36–1.59 Gbp, respectively. This is roughly half the size of the human genome and closer to the smaller genomes of some other sauropsids such as the chicken and the anole lizard (Table 2).

## 3. Genome Data in the Reconstruction of Toxin Evolution

### 3.1. Overview of Possible Mechanisms of Toxin Evolution

Toxin evolution is reviewed in Ref. [32]. Waglerin toxins in Wagler’s viper (*Tropidolaemus wagleri*) may well have arisen de novo since no orthologues have been found [49]. This is an exceptional case and in general, the evolution of genes de novo is thought to be comparatively rare. Thus, in the human genome, entirely new genes (i.e., those not found in other primates) are very few in number, and tend not to be expressed in the proteome, suggesting that they function as non-protein-coding genes [46]. In fact, the likelihood of a gene being expressed in the human proteome at all was found to be related to the age of evolutionary origin of that gene [46].

Cysteine-rich secretory protein (CRISP) and kallikrein toxins in Wagler’s viper are suggested to have become toxic simply as a result of evolutionary changes in the coding sequence of existing salivary proteins [49]. Indeed, another study concluded that not just a few, but in fact most, snake venom toxins evolved from proteins expressed ancestrally in salivary glandular tissue [50]. In any case, it is clear most venom toxins share close sequence similarity, at least in their functional domains, with known, non-venom genes (physiological or body genes) [49].

Alternative splicing can result in both physiological and toxin isoforms being generated from the same gene in different tissues. This appears to be the case with acetylcholinesterase gene of *Bungarus fasciatus* [51].

### 3.2. Moonlighting: The Strange Case of Nerve Growth Factor

Nerve growth factor (NGF) is a component of venoms in many snakes. At first sight, it may seem to be nothing more than an innocuous neurotrophin apparently occurring in the venom for no good reason. However, NGF is an extremely potent inducer of mast cell degranulation; thus it is possible that it may produce increased local vascular permeability and toxin absorption; it may also produce or enhance anaphylaxis [52,53]. The possibility that venom nerve growth factor may contribute to the toxicity of venom is further suggested by the fact that, like other true venom toxins, it is under positive selection in at least some snakes [52,53]. Nerve growth factor may also play an ancillary (non-toxic) role while the venom is stored in the venom gland by inhibiting metalloprotease-mediated degradation [54]. Since a single isoform is present in *Bothrops jararaca* [55] it is possible that nerve growth factor may be ‘moonlighting’ as a venom component—that is, taking on functions in the venom additional to those of its function as a neurotropin (the concept of moonlighting is discussed in Ref. [56]). However, arguing against moonlighting is the fact that nerve growth factor is present in at least two copies in the king cobra genome [3] and in other cobras (reviewed in Ref. [50]; see also Table 3 in the current article).

### 3.3. Gene Duplication

Gene duplication may be important in the evolution of venom toxins at two levels: (i) in the origin of the toxin gene from its ancestral counterpart and (ii) in subsequent expansion of the established toxin gene into a multigene family.

Some toxin genes may have undergone an initial duplication event, after which one copy came to be relatively highly expressed in the venom gland by some change in tissue-specific regulation [32]. The nascent toxin gene could then, in principle, undergo sequence evolution independently of its non-venom paralogue to evolve a new function. This process is called neo-functionalization [57,58]. One problem with neo-functionalization as a mechanism is that mutations are more likely to be deleterious than beneficial [59]. An alternative model of gene evolution after duplication is sub-functionalization. This phenomenon can account for the survival of both paralogues because, weakened in function by deleterious mutations, the two copies will need to be retained in the genome in order to make up, together, the full ancestral function by virtue of their complementary effects [60]. This has been called the duplication-degeneration-complementation (DDC) hypothesis [60].

**Figure 1:**
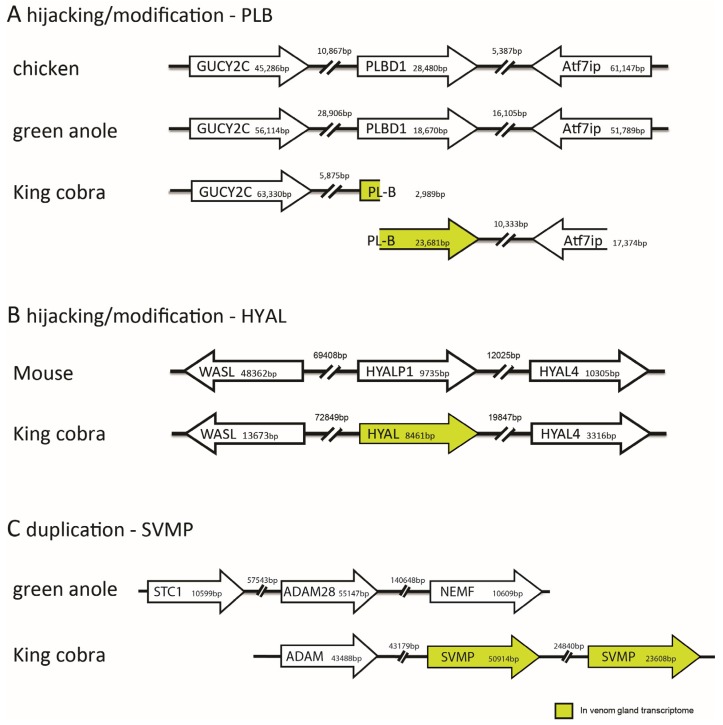
Syntenic comparisons of venom genes in the king cobra with other vertebrates revealing toxin recruitment by hijacking/modification and gene duplication. (**A**) Modification of PLBD1 gene found in the green anole lizard (*Anolis carolinensis*) and the chicken (*Gallus gallus*) results in the venom gland expressed phospholipase-B (PLB). Note that PLB is found split across two king cobra genome scaffolds; (**B**) Modification of HYALP1 gene found in the mouse (*Mus musculus*) results in the venom gland expressed hyaluronidase (HYAL); (**C**) Duplication of the non-venom gland expressed ADAM gene in the king cobra results in a venom gland expressed snake venom metalloproteinase (SVMP) gene. The ADAM gene in the green anole is flanked on both sides by non-SVMP genes, demonstrating the absence of gene duplication in this species. Note that subsequent downstream duplication of the SVMP gene in the king cobra results in multiple venom gland expressed SVMP isoforms. Based on Figure S5 from [3].

There is no predictable pattern of duplication events in toxin evolution, as can be readily seen, for example, in the highly variable number of different toxin paralogues in the king cobra genome (Table 3). The number varies from one (hyaluronidase) to 21 (three-finger toxins). It is possible that some genes have undergone what we have referred to as ‘hijacking’ [3]; that is, sequence modification without duplication (Figure 1 in the current article). Comparative analysis of synteny (Figure 1) suggests that the ancestral PLBD1 gene may have evolved into the king cobra venom phospholipase-B (PLB), and that the HYALP1 gene similarly gave rise to venom-expressed hyaluronidase (HYAL).

An example of a gene that has undergone duplication is the ADAM gene which underwent duplication and subsequently these duplicates evolved into a venom-expressed snake venom metalloproteinase (SVMP) gene (Figure 1). Other toxins that have undergone multiple rounds of duplication to produce multigene families include phospholipase A_2_ in rattlesnakes [5]. In that gene family, some paralogues subsequently disappeared from the genome in different lineages, possibly because of a change in prey type [5]. The origin of genes by duplication, and the subsequent loss of some paralogues in this way, is consistent with the birth-and-death model of the evolution of multigene families [61,62].

### 3.4. Possible Selective Advantage of Possessing Multigene Toxin Families

**Figure 2:**
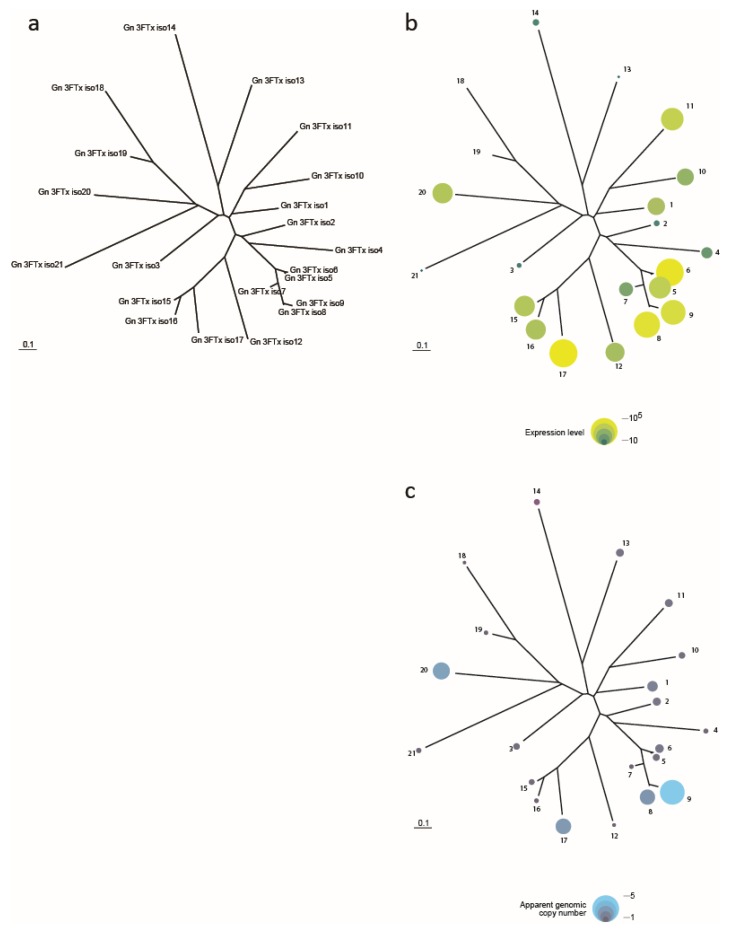
Preliminary analysis of three finger toxin isoforms in the king cobra genome. (**a**) Phylogeny showing isoform numbers; (**b**) Expression level (transcript abundance) in the venom gland; (**c**) Apparent copy number in genome. One hypothesis consistent with the figure is that the more recently expanded paralogues tend to be more highly expressed. The figure is an unpublished analysis by one of us (Christiaan Henkel) based on data in Ref. [3]. See Table 4 for corresponding genome sequencing and accession codes of the three finger toxin isoforms.

A preliminary analysis of the king cobra genome (Figure 2) suggests one possible selective advantage of duplication in the evolution of multigene toxin families. There is a tendency for paralogues that have undergone recent expansion to be more highly expressed in the venom gland transcriptome. More work is required to confirm this hypothesis although it is consistent, for example, with the relationship between amylase abundance and mRNA abundance in mice [63].

Other explanations for the evolution of multiple isoforms of the same toxin is that they might provide broad spectrum toxicity against a range of prey species. Presumably, this is more likely to be advantageous for generalists, than for specialists such as the king cobra. Multiple isoforms might also provide potentiation, so that the toxin complex is more potent than a single toxin. Potentiation is known, for example, in the cone snails [64]. The possession of multiple gene copies might make it more difficult for prey to evolve resistance.

### 3.5. The Selective Expression of Toxin Genes, or Their Ancestral Orthologues, in the Venom Gland

Given that many toxins have arisen by duplication from genes whose ancestral function was something other than that of a venom toxin [5,49,55,65], how did one or more of the duplicates (paralogues) come to be selectively expressed in the venom gland?

#### 3.5.1. Recruitment and Neo-Functionalisation Hypothesis

One scenario for the selective expression of toxin genes in the venom gland is as follows [49]. One of the copies of the ancestral gene underwent a change in tissue-specific regulation so as to become expressed de novo in the venom gland [66]. This paralogue then underwent evolution of its coding sequence so as to become more effective as a venom toxin [3]. Such adaptive changes in the coding sequence of the newly-recruited gene represent “neo-functionalization”—the evolution of a function not related to the ancestral function (reviewed in Ref. [57,58]). Changes in the coding sequence may be accompanied by additional changes in the regulation of toxin gene transcription, as well as in translation and post-translational modification of the protein [67]. Finally, there is evidence that a toxin gene may ultimately undergo a further change in tissue-specific regulation and revert to being expressed in a tissue or organ other than the venom gland [55,68]. While this hypothesis has been disputed [69], recent work comparing toxin expression in multiple different tissues of *B. jararaca* provided additional evidence for reverse recruitment [55].

#### 3.5.2. Restriction and Sub-Functionalisation Hypothesis

The hypothesis of duplication and neo-functionalisation outlined above has been questioned [50] on the grounds that gene duplication in vertebrate genomes is an extremely rare event, and that persuasive examples of neo-functionalisation have rarely been described in any context. Furthermore, since new transcriptional regulatory relations have to be established for a gene to become highly expressed in the venom gland, the whole scenario is argued to be improbable [50].

An alternative hypothesis is that the ancestral gene was expressed in a wide range of tissues, including the venom gland; it then underwent duplication, with one paralogue becoming restricted in expression to the venom gland and losing expression in the other tissues [50]. Thus, while the recruitment and neo-functionalisation hypothesis is critically dependent on the acquisition of new regulatory regions (for the novel expression of a paralogue in the venom gland), the recruitment and sub-functionalisation depends on the loss of regulatory regions (that ancestrally drove expression in tissues other than the venom gland).

#### 3.5.3. Testing the Recruitment and Restriction Hypotheses

It may well prove to be difficult or impossible to test these hypotheses using comparative transcriptomic data only. An essential pre-requisite will be the availability of multiple snake genomes that provide appropriate taxon sampling, together with the identification of regulatory regions that control the tissue-specific expression of toxin genes and their ancestral paralogues. Putative regulatory sequences will also have to be tested functionally. Progress is being made in this area, as we shall now discuss.

### 3.6. Mechanisms of Transcriptional Regulation That Might Have Led to Selective Expression of Toxin Genes in the Venom-Gland

#### 3.6.1. Non-Coding RNA Genes

RPTLN are long, non-coding RNA genes that may have been involved in the evolution of snake venom metalloproteinases (SVMPs) from a disintegrin and metalloproteinase (ADAM) gene. According to one hypothesis [66], RPTLN was under the control of a venom gland promotor and its signal sequence became fused with the extracellular domain of the one copy of the ancestrally physiological ADAM gene (the latter having previously undergone tandem duplication). After thus being activated in the venom gland, the ADAM gene evolved into an SVMP [66]. The authors note that their hypothesis can be tested as soon as genome builds for the relevant snake species are available (see also this issue: see Ref. [70] for more information).

#### 3.6.2. Transposable Elements

Another intriguing possibility is that *CR1 LINE* transposable elements, which are much more abundant in advanced than in basal snakes, may have played a role in toxin gene recruitment [2]. *CR1 LINEs* are abundant in the genome of the copperhead (*Agkistrodon contortrix*)—much more abundant than they are in the Burmese python (*Python molurus bivittatus*) genome [41]. We discuss transposable elements and other repetitive sequences in more detail below.

#### 3.6.3. VERSE

It has previously been shown that the gene sequences of TroD (venom prothrombin activator gene) and TrFX (blood coagulation factor X gene) are highly similar, except for promoter and intron 1 regions, indicating that TroD probably evolved by duplication of its plasma counterpart [22]. The insertion, in the promoter of TroD, of a VERSE sequence (VEnom Recruitment/Switch Element) accounts for elevated, but not tissue-specific, expression [23].

#### 3.6.4. AG-Rich Motifs

More recently, it was found that AG-rich motifs, in the first intron, silence gene expression in non-venom gland tissues [71]. These AG-rich motifs are promotor-independent silencers, and such cis-elements are also found in some snake toxin genes, but not in housekeeping genes. Several polycomb group proteins (transcription factors) were identified to bind these motifs to regulate expression. Genome sequences will help in identifying regulatory elements that control tissue-specific expression of toxin genes in venom glands as well as expression of cognate genes in respective tissues.

### 3.7. Evolution of Toxin Resistance in Snakes as Studied with Genomic Data

Genome sequences have cast light on the resistance by snakes to the toxins of their prey. Thus, the Eastern hog-nosed snake (*Heterodon platirhinos*) is resistant to the tetrodotoxin of *Notophthalmus viridescens* a newt on which it preys [72], and the garter snake *Thamnophis sirtalis* likewise shows resistance to the neurotoxic tetrodotoxin of newts in the genus *Taricha* [73]. Tetrodotoxin resistance in *Thamnophis* is due to modification of the amino acid sequence of tetrodotoxin targets: sodium ion channels on skeletal muscles (Na_v_1.4) and peripheral neurons (Na_v_1.6 and Na_v_1.7; reviewed in Refs. [73,74]). Analysis of snake genomic data and partial sequences, suggests that tetrodotoxin resistance in *Thamnophis* arose stepwise over a long period of evolutionary time, with the ancient modifications of the sodium channels in nerves providing the necessary conditions for evolution of resistance in skeletal muscle sodium-channels [73].

## 4. Transposable Elements and Other Repetitive Sequences in Snake Genomes

Repetitive elements (repeats) are DNA sequences present in many copies in a genome; they can be classified into tandem repeats and transposable elements [75]. They are relatively abundant in snake genomes, especially the genomes of advanced snakes.

Studies of snake genomes have shown how transposable elements have accumulated in the Hox complex of developmental genes. The Hox complex consists of transcription factor genes that have important roles in regulating embryonic pattern formation. Di-Poï and colleagues selectively sequenced the 5′ regions of the Hox clusters of different species [6]. In the squamates studied, including the corn snake (*Pantherophis guttatus*) the clusters had become expanded in size due the accumulation of numerous transposable elements. These transposons include retrotransposons and DNA transposons, and occur mainly in the introns and intergenic regions [6]. The availability of genomic sequences is also helping to uncover possible incidences of horizontal gene transfer. Thus, the long interspersed element (LINE) non-LTR retrotransposon BovB is suggested to have been transferred, by tics, from squamates to bovids [28].

## 5. Future Prospects in Snake Genomics

In the future, it may be possible to scan snake genomes for bioactive molecules; this could be done, for example, by comparison with a pharmacophore database. Drug discovery from venoms has already delivered drugs such as Prialt, Integrilin, Captopril and Byetta, and multiple candidates are now progressing in clinical trials [76,77]. Furthermore, peptides derived from venoms are valid pharmacological tools to study diseases [78]. For example, the study of the snake toxin α-bungarotoxin led to characterisation of the nicotinic acetylcholine receptor (nAChR) and a new understanding of the disease myasthenia gravis [79]. Genome sequences also provide the prospect of generating recombinant antivenoms [80]. Additionally, methylome sequencing will allow us to investigate the role of epigenetics in regulating toxin gene expression [81].

Another step forward in snake genome sequencing will be new sequencing techniques. Next- or second-generation sequencing, so-called because of the advance in Sanger sequencing, produces short reads with low error rates and high throughput [82]. Examples of next-generation platforms are Illumina and Roche 454. The newly-emerging third-generation sequencing platforms are able to provide reads many kB long [83,84]. These platforms include the so-called “PacBio” system—single molecule, real-time (SMRT) sequencing—from Pacific Biosciences; and MinION™ from Oxford Nanopore Technologies [82]. MinION™ has a higher error rate than PacBio [82] and both have higher error rates than second-generation sequencing. For a review of different sequencing platforms and their properties, see Ref. [84].

Given the relatively high percentage of repetitive sequences in advanced snake genomes, a hybrid approach is very promising, and has indeed proved useful for the tackling the same problem in the human genome [84]. This approach involves using the long reads of third-generation sequencing to bridge the gaps due to repeats; and combining them with reads from second-generation sequencing to ameliorate the problem of errors in the long reads [84]. We have used this approach to assemble a draft genome of the Malayan pit viper (unpublished data). However, as the error rate of long-read sequencing improves, the hybrid approach will likely lose favor.

Other interesting developments in sequencing include optical mapping [85], useful for examining the large-scale organization of genomic features, especially around large repetitive clusters; and single-cell RNA-seq [86]. The latter would be very useful in investigating the regulation of toxin production in the venom gland, as it provides a transcriptomic profile per individual cell (and thereby an overview of the different cell types in a gland).

## Figures and Tables

**Table 1:** Snake genome projects published or in progress.

Trivial Name	Scientific Name	Family	Notes
Prong-snouted blind snake	*Anilios bituberculatus*	Typhlopidae	F.J. Vonk et al., in progress
Texas blind snake	*Rena dulcis*	Leptotyphlopidae	T.A. Castoe et al., in progress
Boa constrictor	*Boa constrictor*	Boidae	Ref. [13]; GenB: PRJNA210004
Boa constrictor	*Boa constrictor*	Boidae	Ref. [14]
Burmese python	*Python bivittatus*	Pythonidae	Published [2]; GenB: AEQU00000000
Garter snake	*Thamnophis sirtalis*	Colubridae	GenB: LFLD00000000
	*Thamnophis elegans*	Colubridae	Ref. [13]; GenB: PRJNA210004
Corn snake	*Pantherophis guttatus*	Colubridae	Ref. [15]; GenB: JTLQ01000000
Corn snake	*Pantherophis guttatus*	Colubridae	Targeted sequencing: 5′ hox genes [16]
King cobra	*Ophiophagus hannah*	Elapidae	Published [3]; GenB: AZIM00000000
Malayan pit viper	*Calloselasma rhodostoma*	Viperidae	F.J. Vonk et al., in progress
Five-pacer viper	*Deinagkistrodon acutus*	Viperidae	Ref. [17]
European adder	*Vipera berus berus*	Viperidae	Baylor College of Medicine, Human Genome Sequencing Center; GenB: JTGP00000000
Habu	*Protobothrops flavoviridis*	Viperidae	H. Shibata et al., in progress
Brown spotted pit viper	*Protobothrops mucrosquamatus*	Viperidae	A.S. Mikheyev et al., in progress; GenB: PRJDB4386
Prairie rattlesnake	*Crotalus viridis viridis*	Viperidae	T.A. Castoe et al., in progress
Western diamond-backed rattlesnake	*Crotalus atrox*	Viperidae	Ref. [5]
Timber rattlesnake	*Crotalus horridus*	Viperidae	GenB: LVCR00000000.1
Speckled rattlesnake	*Crotalus mitchellii pyrrhus*	Viperidae	Ref. [18]; GenB: JPMF01000000
Western Diamondback rattlesnake, Mojave rattlesnake and Eastern Diamondback rattlesnake	*Crotalus atrox, C. scutulatus, and C. adamanteus*	Viperidae	Targeted sequencing of bacterial artificial chromosome (BAC) clones containing phospholipase A_2_ genes.
Pygmy rattlesnake	*Sistrurus miliarius*	Viperidae	Ref. [13]; GenB: PRJNA210004
Temple pit viper	*Tropidolaemus wagleri*	Viperidae	R.M. Kini et al., in progress

This list is not necessarily exhaustive. Abbreviation: GenB, GenBank accession number. Taxonomy according to the Pubmed Taxonomy database [19].

**Table 2:** Selected data from the Burmese python and king cobra draft genomes and comparison with genomes of other species.

Species	Coding Genes (k)	Genome Size (Gb)	Repeats (%)
Burmese python	25 [2]	1.44 [2]	31.8–59.4 [2]
King cobra	21.19 [3]	1.36–1.59 [3]	35.2–60.4 [2]
Chicken	20–23 * [44]	1.05 [44]	4.3–8.0 [45]; 9.4 [44]
Human	20.4 ^¶^; 19 [46]	3.54 ^¶^	>66–69 [47]
Anolis	18.5 ^†^	1.70 ^†^	30% ^‡^ [48]

* v. 85.4 in ensembl.org gives the number of coding genes in the chicken genome as 15,508; ^¶^ Human genome, build 38; ensembl.org; ^†^ GenBank Assembly ID GCA_000090745.1; ^‡^ Mobile elements.

**Table 3:** Number of copies (paralogues) of toxin genes in the king cobra (*Ophiophagus hannah*) genome; data from Ref. [3].

Venom Toxin or Toxin Family	Number of Paralogues
3FTx (three-finger toxin) *	21
PLA_2_ (phospholipase A_2_) *	12
Lectin *	11
Kunitz *	10
Waprins *	6
Cystatin	5
CRISP (cysteine-rich secretory protein)	3
CVF (cobra venom factor)	3
Kallikrein	3
SVMP (snake venom metalloproteinase)	3
LAAO (L-amino acid oxidase)	2
NGF (nerve growth factor)	2
NP (natriuretic peptide) *	2
Acetylcholinesterase	1
Hyaluronidase	1
PLB (phospholipase-B)	1
VEGF (vascular endothelial growth factors)	1
Vespryn	1

Key: (*) estimated number of paralogues; the current genome assembly is not sufficiently well-scaffolded to allow the number of paralogues to be determined with certainty.

**Table 4:** King cobra three finger toxin genome sequencing and accession codes. Isoforms correspond with the ones referred to in Figure 2.

3FTX Isoform	Nucleotide Sequence	Accession Code Genbank
Iso1	GATACACCTTGACATGTCTAACACATGAATCATTATTTTTTGAAACCACTGAGACTTGTTCAGATGGGCAGAACCTATGCTATGCAAAATGGTTTGCAGTTTTTCCAGGTG	AZIM01011044.1
Iso2	GATACACCAGGATATGCCACAAATCTTCTTTTATCTCTGAGACTTGTCCAGATGGGCAGAACCTATGCTATTTAAAATCGTGGTGTGACATTTTTT	AZIM01016929.1
Iso3	GATACACCTTGACATGCATCACATCTGCTCGTAACTTTGAGACTTGTCCACCTGGGCAGAACCTATGCTTTTTAAAATCATGGTATGAAGCTTCAT	AZIM01214498.1
Iso4	TACAAAACCGGTGAACGTATTATTTCTGAGACTTGTCCCCCTGGGCAGGACCTATGCTATATGAAGACTTGGTGTGACGTTTTTT	AZIM01146344.1
Iso5	GATACACCATGACATGTTACACACAGTACTCATTGTCTCCTCCAACCACTAAGACTTGTCCAGATGGGCAGAACCTATGCTATAAAAGGTGATTTGCGTTTATTCCACATG	AZIM01015434.1
Iso6	GATACACCACGAAATGCTACGTAACACCTGATGCTACCTCTCAGACTTGTCCAGATGGGGAGAACATATGCTATACAAAGTCTTGGTGTGACGGTTTTT	AZIM01133918.1
Iso7	GATACACCACGAAATGCTATGTAACACCTGATGCTACCTCTCAGACTTGTCCAGATGGGGAGAACATATGCTATACAAAGTCTTGGTGTGACGTTTTTT	AZIM01229389.1
Iso8	GATACACCACGAAATGCTACATAACACCTGATGTGAAGTCTCAGACTTGTCCAGATGGGGAGAACATATGCTATACAAAGACTTGGTGTGATGTTTGGT	AZIM01229389.1
Iso9	GATACACCACGAAATGCTACGTAACACCTGATGTTAAGTCTGAGACTTGTCCAGATGGGCAGGACATATGCTATACAGAGACTTGGTGTGACGTTTGGT	AZIM01028336.1
Iso10	GATACACCACGAAATGCTACGTAACACCTGATGTTAAGTCTGAGACTTGTCCAGCTGGGCAGGACATATGCTATACAGAGACTTGGTGTGATGCTTGGT	AZIM01097792.1
Iso11	GACACACCAGGATATGTCTCACAGACTACTCAAAAGTTAGTGAAACCATTGAGATTTGTCCAGATGGGCAGAACTTCTGCTTTAAAAAGTTTCCTAAGGGTATTCCATTTT	AZIM01006046.1
Iso12	GATACACCATGAAATGTCTCACAAAGTACTCCCGGGTTAGTGAAACCTCTCAGACTTGTCACGTTTGGCAGAACCTATGTTTTAAAAAGTGGCAGAAGG	AZIM01011575.1
Iso13	GACACACCTTGATATGTGTCAAACAGTACACAATTTTTGGTGTAACCCCTGAGATTTGCGCAGATGGGCAGAACCTATGCTATAAAACATGGCATATGGTGTATCCAGGTG	AZIM01011969.1
Iso14	GATACACCACGAAATGTTACAACCACCAGTCAACGACTCCTGAAACCACTGAAATTTGTCCAGATTCAGGGTACTTTTGCTATAAAAGCTCTTGGATTGATGGACGTG	AZIM01034614.1
Iso15	GATACACCCTGATATGTCACCGAGTGCATGGACTTCAGACTTGTGAACCAGATGAGAAGTTTTGCTTTAGAAAGACGACAATGTTTTTTCCAAATC	AZIM01009352.1
Iso16	GATACACCAGGAAATGTCTCAACACACCGCTTCCTTTGATCTATANTTAAAATGACTATTAAGAAGTTGCCATCTA	AZIM01009586.1
Iso17	NATACACCAGGATATGTTTAAAGCAAGAGCCATTTCAACCTGAAACCAGTACAACTTGTCCAGATGGGGAAGATGCTTGCTATAGTACATTTTGGAGTGATAACC	AZIM01019523.1
Iso18	NATACACCAGGATATGTTTAAAGCAAGAGCCGTTTCAACCTGAAACCACTACAACTTGTCCAGAAGGGGAGGATGCTTGCTATAATTTGTTTTGGAGTGATCACA	AZIM01052732.1
Iso19	GATACAGCTTGATATGTTTTAACCAAGAGACGTATCGACCTGAAACCACTACAACTTGTCCAGATGGGGAGGACACTTGCTATAGTACATTTTGGAATGATCACCATG	AZIM01009977.1
Iso20	CACAAACCAAGACATGTTACTCATGCACTGGAGCATTTTGTTCTAATCGTCAAAAATGTTCGGGTGGGCAGGTCATATGCTTTAAAAGTTGGAAAAATACTCTTCTGATAT	AZIM01013260.1
Iso21	CACACACCCTGACATGTTACTCATGCAATGGATTATTATGTTCTGACCGTGAACAATGTCCAGATGGGTAGGACATATGCTTTAAGAGATGGAATGATACTGATTGGTCAG	AZIM01013561.1
Iso22	GATACAGCTTGACATGTCTCAATTGCCCAGAACAGTATTGTAAAAGAATTCACACTTGTCGAGATGGGGAGAACGTATGCTTTAAAAGGTTTTACGAGGGTAAACTATTAT	AZIM01071124.1
Iso23	GATACACTCTGTTGTGTTGCAAATGCAATCAAACGGTTTGTGATCTCAATTCGTATTGTTCAGCAGGCAAGAACCAATGCTATATATTGCAGAATAATA	AZIM01008565.1
